# Supplementary material for: Vemurafenib Inhibits Enterovirus A71 Genome Replication and Virus Assembly
Source: Pharmaceuticals (Basel). 2022 Aug 27;15(9):1067. doi: 10.3390/ph15091067 (PMC9500672; doi:10.3390/ph15091067)
Supplement: Supplementary file 1 [file pharmaceuticals-15-01067-s001.zip › pharmaceuticals-1848841-supplementary.pdf]

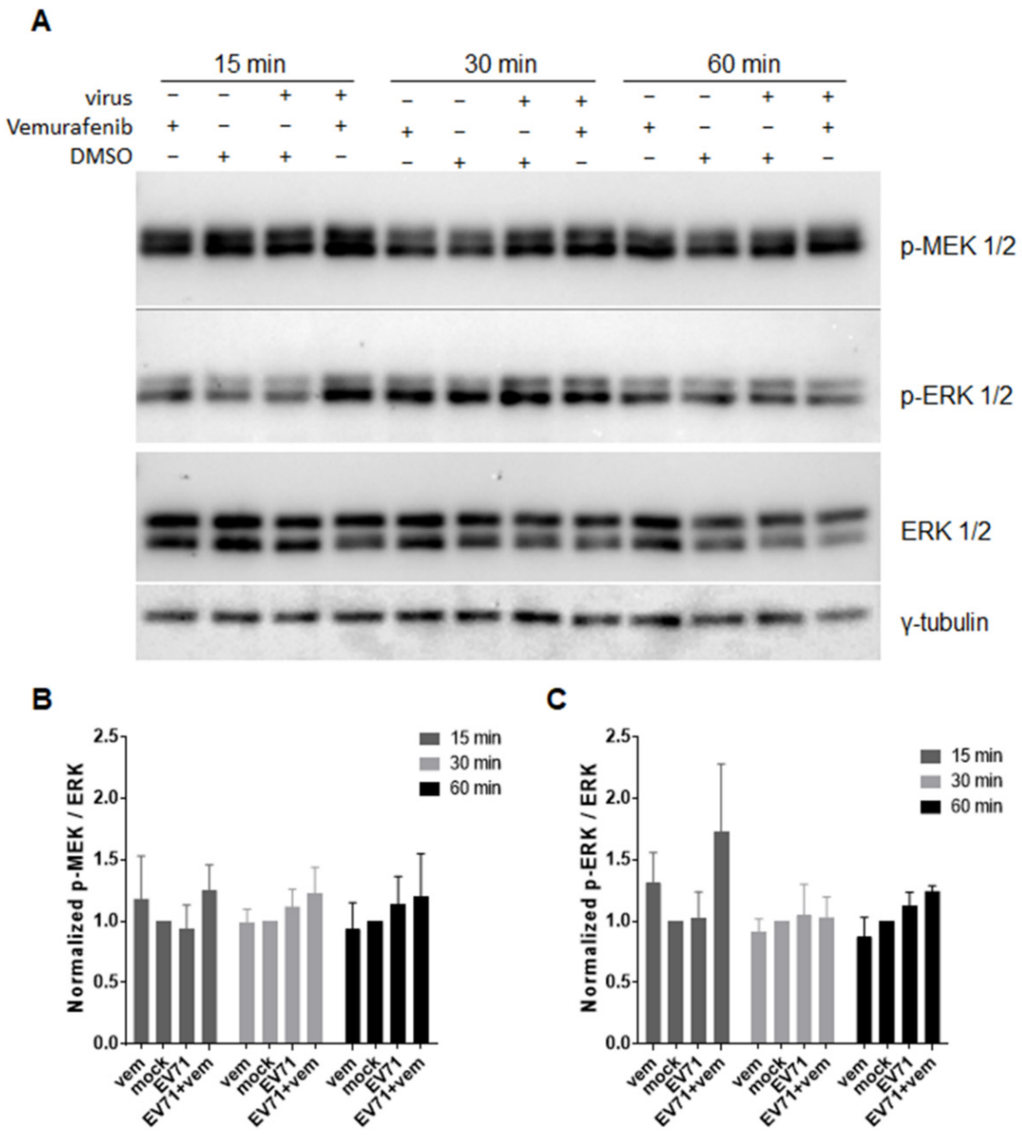

Figure S1. Responses of RAF-MEK-ERK kinase cascade to vemurafenib treatment at earlier time points of EV-A71 infection. **(A-C)** RD cells were infected without or with EV-A71 at an MOI of 5 and concurrently treated with 4 μM vemurafenib or 0.02% DMSO for 1h. Cells were then washed once with DMEM and replenished DMEM. Cell lysates were collected at 15min, 30 min, and 60min post infection. Activation of RAF-MEK-ERK kinase cascade was analyzed by western blot. Phosphorylated MEK1/2 and ERK1/2 were detected using anti-phospho-MEK1/2 (Ser217/221) antibody and anti-phospho-ERK1/2 (Thr185/187) antibody. Unphosphorylated ERK and γ-tubulin were detected as controls. Vem: cells were mock infected and treated with 4 μM vemurafenib. Mock: cells were mock infected and treated with 0.02% DMSO. EV71: cells were infected with EV71 at an MOI of 5 and treated with 0.02% DMSO. EV71 + vem: cells were infected with EV71 at an MOI of 5 and treated with 4 μM vemurafenib. Blots are representative data of three independent experiments. (B+C) Quantification of relative amount of phosphorylated MEK 1/2 (B) or phosphorylated ERK 1/2 (C) to unphosphorylated ERK and normalized to mock for each time point. Results from three independent experiments are displayed as means ± standard deviation.
